# Impact of programmed death‐ligand 1 (PD‐L1) positivity on clinical and molecular features of patients with metastatic gastric cancer

**DOI:** 10.1002/cam4.6472

**Published:** 2023-09-01

**Authors:** Minkyue Shin, Soomin Ahn, Jaeyun Jung, Sujin Hyung, Kyoung‐Mee Kim, Seung Tae Kim, Won Ki Kang, Jeeyun Lee

**Affiliations:** ^1^ Division of Hematology‐Oncology, Department of Medicine Sungkyunkwan University School of Medicine, Samsung Medical Center Seoul South Korea; ^2^ Department of Pathology and Translational Genomics Sungkyunkwan University School of Medicine, Samsung Medical Center Seoul South Korea; ^3^ Innovative Institute for Precision Medicine, Samsung Medical Center Seoul South Korea

**Keywords:** combined positive score, Epstein–Barr virus, gastric cancer, immune checkpoint inhibition, microsatellite instability, next‐generation sequencing, programmed death‐ligand 1, tumor mutational burden

## Abstract

**Background:**

Programmed death‐ligand 1 (PD‐L1) is an important screening biomarker to select patients with gastric cancer (GC) for optimized treatment, including immune checkpoint inhibitors (ICI).

**Methods:**

In this single‐institution retrospective cohort study, patients with metastatic GC with available PD‐L1 results between October 2019 and September 2021 were identified by reviewing their electronic medical records. Genomic data were obtained from the Samsung Medical Center Clinical Sequencing Platform.

**Results:**

Among the 399 patients, 276 (69%) had a PD‐L1 combined positive score (CPS) ≥1, 155 (39%) had a CPS between 1 and 5, and 121 (30%) had a CPS ≥5. Of the 121 patients with CPS ≥5, 28 (23%) had a known etiology for “inflamed tumor,” with Epstein–Barr virus (EBV) positivity (*N* = 11) or high tumor mutational burden (TMB) (*N* = 17), which included microsatellite instability (MSI) (*N* = 9). PD‐L1 CPS ≥5 was observed in 11/11 (100%) patients with EBV positivity, 9/12 (75%) patients with MSI, and 17/33 (52%) patients with high TMB. For the 108 patients who received ICI therapy, CPS ≥5 was the only predictor significantly associated with survival in multivariable analyses, including TMB, MSI, or EBV. Objective response rate (ORR) was 49% in patients with CPS ≥5, 30% in patients with 1 ≤ CPS <5, and 19% in patients with CPS <1. Among the 31 responders to ICI therapy, 27 (87%) had a CPS of ≥1. Mutations in *TET2*, *IRS2*, *DOT1L*, *PTPRT*, and *LRP1B* were associated with a higher ORR (63%–100%), whereas *MDC1* mutations were associated with a low ORR (22%).

**Conclusions:**

PD‐L1 expression is an independent and sensitive biomarker for ICI therapy. Considering its significant association with several gene alterations, including *PIK3CA* mutations and *MET* amplification, combining ICI therapy with other targeted agents may be a promising therapeutic strategy for GC.

## 1 INTRODUCTION

Gastric cancer (GC) is the fourth leading cause of cancer‐related deaths and the fifth most common malignancy worldwide.^1^ In landmark phase III trials, Checkmate‐649 and KEYNOTE‐590,^2^, ^3^ the addition of immune checkpoint inhibitors (ICIs) to chemotherapy as a first‐line treatment has demonstrated survival benefits in GC, and gastroesophageal and esophageal adenocarcinoma. In KEYNOTE 811 trial,^4^, ^5^ the addition of pembrolizumab and trastuzumab to chemotherapy resulted in extended survival. Expression of programmed death‐ligand 1 (PD‐L1) is measured by a combined positive score (CPS), which predicts which individual would likely benefit from ICIs: it is ≥5 for nivolumab and ≥ 10 for pembrolizumab.^6^ In addition, microsatellite instability (MSI) and Epstein–Barr virus (EBV) positivity have been reported as mutually exclusive biomarkers for response to pembrolizumab in a phase II clinical trial.^7^ With the recent approval of ICIs for the treatment of GC by the United States Food and Drug Administration (US FDA), the importance of PD‐L1 has been underscored in terms of its clinical utility in the oncology community.

GC is a heterogeneous disease that requires in‐depth molecular analyses for the development of personalized medicine.^8^, ^9^ Targeted therapy guided by prospective clinical sequencing demonstrated better outcomes than conventional chemotherapy in an umbrella trial, especially with *MET* amplification and *PIK3CA* mutation.^10^ Another study performed targeted next‐generation sequencing (NGS) of 295 patients with metastatic GC, including 40 patients treated with ICIs, to identify predictive biomarkers for systemic therapies.^11^ In addition, previous NGS studies of melanoma and non‐small cell lung carcinoma have demonstrated a significant association between tumor mutational burden (TMB) and benefits from ICIs, also revealing specific gene alterations associated with the response to ICIs.^12^, ^13^ Recent NGS studies in GC also demonstrated a significant association between TMB and benefit from ICIs, but only identified a single gene alteration (*PIK3CA* mutation) that was significantly associated with the ICI response.^14^, ^15^, ^16^ The limited number of patients and genes assessed in the above studies may have led to a lack of understanding of GC heterogeneity. Given the importance of PD‐L1 (+) GCs in the clinic, it is important to understand the molecular and clinical characteristics of PD‐L1 (+) GC tumors.

Here, we describe PD‐L1 (+) GC tumors in terms of molecular and pathological traits, such as EBV positivity, MSI status, and TMB, as screened by clinical NGS. The aim of this study was to understand the clinical implications of the PD‐L1 immunohistochemistry (IHC) assay used as part of clinical practice for the majority of patients with GC seen in oncology clinics. We present real‐world evidence that the PD‐L1 assay predicts the response to ICIs independent of the other aforementioned biomarkers, even though they are closely related to each other. In addition, we identified six gene alterations significantly associated with ICI response, and dozens of gene alterations were significantly enriched in patients with PD‐L1 (+) GC, which were not identified in previous studies. Notably, these included some clinically actionable genes, such as *MET* and *PIK3CA*, which could be potential combinatorial therapeutic targets.

## 2 MATERIALS AND METHODS

**FIGURE 1:**
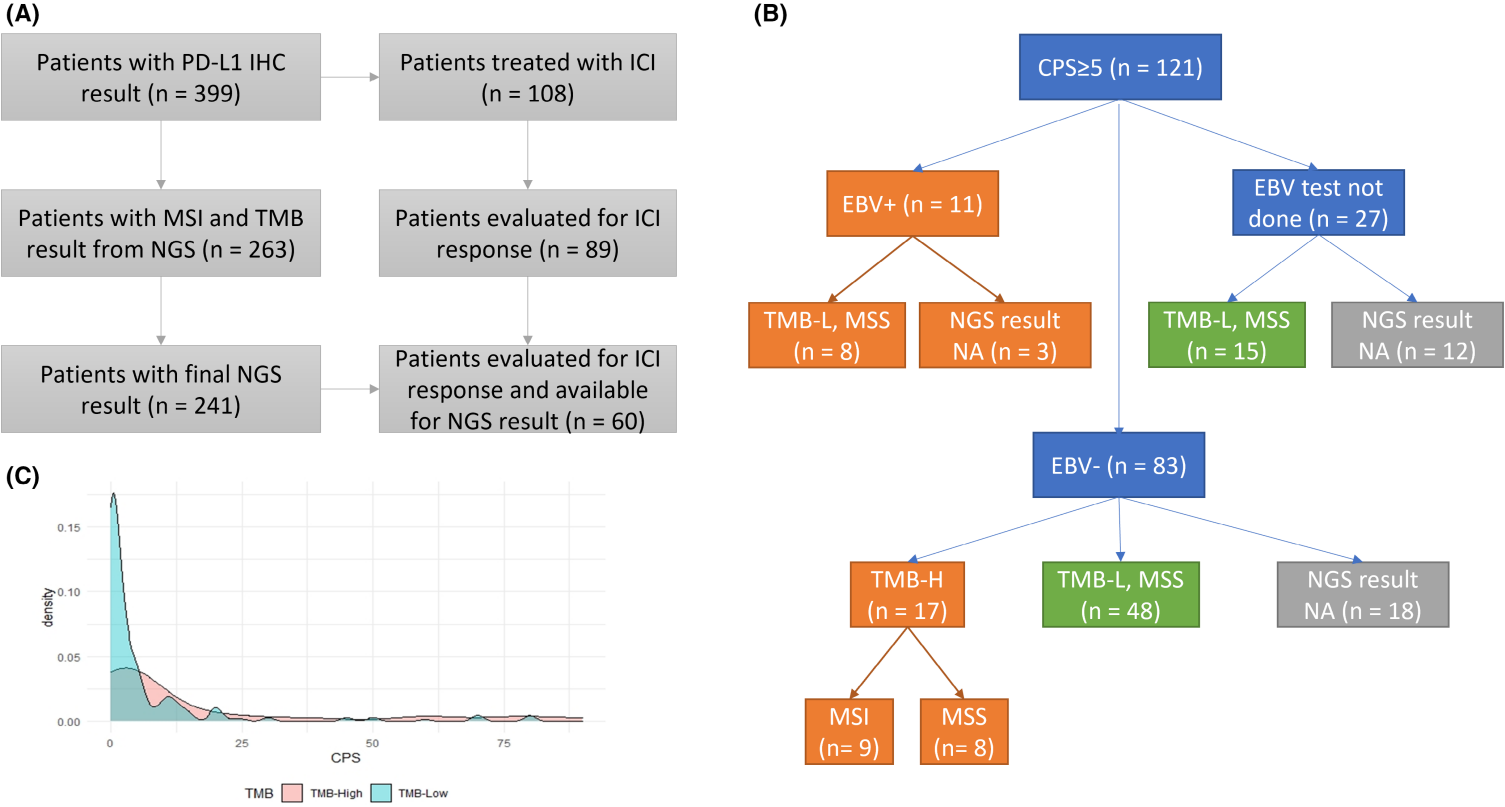
Patient consort diagram of (A) overall patients and (B) patients with a CPS of 5 or greater. (C) Density plot showing CPS distribution according to TMB status (*n* = 263).

In this study, we recruited 399 consecutive patients with stage IV GC admitted to the Samsung Medical Center (Seoul, Korea) between October 2019 and September 2021, who were tested using PD‐L1 IHC prior to their treatment. Pathology was confirmed by either primary or metastatic tumor biopsy, and clinical staging was determined using either computed tomography or magnetic resonance imaging. Five patients whose PD‐L1 expression values were not reported in a numeric form, and were reported as high, low, or positive, were excluded from the study. We obtained the following clinicopathological data from medical records: age, sex, smoking history, HER2 and EBV status, treatment regimen, best response, progression date, and survival. NGS results were reported for 241 patients as of November 1st, 2021 (Figure 1). This retrospective study was approved by the Institutional Review Board of Samsung Medical Center (Seoul, Korea). First‐line (1 L) chemotherapy mainly consisted of fluoropyrimidine plus platinum; the detailed regimens are described in Table S1. In addition, we identified patients who had received anti‐PD‐1 or anti‐PD‐L1 agents, that is ICIs, including pembrolizumab, nivolumab, durvalumab, and atezolizumab.

PD‐L1 IHC was performed using monoclonal mouse anti‐PD‐L1 clone 22C3 (Agilent Technologies) with a DAB IHC Detection kit on a BenchMark Ultra (Ventana). The CPS was calculated by counting the number of PD‐L1‐stained cells (tumor cells, lymphocytes, and macrophages), dividing by the total number of viable tumor cells, and multiplying by 100. HER2 IHC was performed using a monoclonal rabbit PATHWAY anti‐HER2/neu (Ventana).

EBV status was determined using EBV‐encoded small RNA in situ hybridization. NGS was performed using the TruSight Oncology 500 (TSO500) assay (Illumina), as described previously.^17^ The panel includes multiple variant types across 523 genes and enables quantitative assessment of MSI and TMB status. TSO500 interrogates 130 MSI loci to calculate total count of unstable sites (total MSI), and MSI was defined as ≥10 total MSI. Total TMB was determined as mutations per megabase (mt/mb) sequenced, and TMB‐high was defined as ≥10 total TMB. Genomic variants were stratified using a four‐tier system based on clinical significance proposed by the Association for Molecular Pathology, American Society of Clinical Oncology, and College of American Pathologists.^18^

Treatment response was evaluated according to RECIST 1.1, and the best response was assessed for the treatment period within the same regimen. The objective response rate (ORR) was defined as the percentage of patients with a complete response (CR) or partial response (PR). Disease control rate (DCR) was defined as the percentage of patients with CR, PR, or stable disease (SD). Progression‐free survival (PFS) was defined as the period from the date of ICI treatment initiation to the date of progressive disease (PD). Overall survival (OS) was defined as the period from the date of ICI treatment initiation to death. Kaplan–Meier method and log‐rank test in the “survival” R library were used to compare PFS and OS between subgroups. Patients who did not experience an event of interest were censored on the date of last follow‐up.

The associations of CPS with clinical and molecular characteristics and treatment responses were analyzed using the Cochran–Armitage trend test. It modifies the Pearson's chi‐square test to assess the linearity between a continuous variable with k categories and variable with two categories. Associations between ICI response and categorical molecular characteristics were analyzed using the Pearson's chi‐square test or Fisher's exact test. The expected effect size of a chi‐square test was calculated using the “pwr” R library. Variables of the logistic regression model predicting ICI response were selected by Akaike information criterion in a stepwise algorithm using the “stats” R library. Prediction of ICI response with quantitative molecular markers was conducted using area under the receiver operating characteristic curve (AUROC) analysis in the “pROC” R library. Oncoprints were plotted using the “ComplexHeatmap” R library, which adapts the “memo sort” method to reorder columns. The gene alterations depicted in the plot were selected based on their frequencies across all samples. The “survivalAnalysis” R library was used to fit Cox proportional hazards regression models and draw forest plots. All statistical analyses were performed using R version 4.0.3 (R Foundation for Statistical Computing, Vienna, Austria).

## 3 RESULTS

### 3.1 Clinicopathologic characteristics of PD‐L1 (+) GC

Between October 2019 and September 2021, 399 patients with metastatic GC had available PD‐L1 IHC results for analysis. A consort diagram is shown in Figure 1A, and baseline clinical and molecular characteristics are presented in Table 1. Overall, 276 patients (69%) had a PD‐L1 CPS ≥1, 155 (39%) had a CPS between 1 and 5, and 121 (30%) had a CPS ≥5. Of the 121 patients with CPS ≥5, 28 had a known etiology for “inflamed tumor” such as EBV‐positive (*N* = 11) or TMB‐high tumor (*N* = 17), which included MSI tumor (*N* = 9). PD‐L1 CPS ≥5 was observed in 11/11 (100%) patients with EBV positivity, 9/12 (75%) patients with MSI, and 17/33 (52%) patients with high TMB. By contrast, 48 patients with CPS ≥5 were non‐EBV‐positive, microsatellite stable (MSS), and TMB‐low (Figure 1B). The underlying mechanisms of these inflamed GC tumors are yet to be determined. In a density plot between the TMB score and PD‐L1 CPS (Figure 1C), most of the TMB‐low patients with GC had a PDL1 CPS of 0 or 1, whereas some TMB‐high patients had a very high CPS (20–80). Furthermore, MSI tumors are a subset of TMB‐high tumors, whereas EBV‐positive tumors are mutually exclusive of TMB‐high or MSI tumors.

**TABLE 1:** Clinicopathologic characteristics of patients included in this study.

Characteristic	Sample size, *n*	CPS <1, *n* (%)	1 ≤ CPS <5, *n* (%)	5 ≤ CPS, *n* (%)	*p*‐Value
All patients	399	123	155	121	
Age					
<65 years	286	96 (78.0%)	106 (68.4%)	84 (69.4%)	0.1333
≥65 years	113	27 (22.0%)	49 (31.6%)	37 (30.6%)	
Sex					
Male	249	68 (55.3%)	95 (61.3%)	86 (71.1%)	0.0110
Female	150	55 (44.7%)	60 (38.7%)	35 (28.9%)	
Smoking					
Current/Ex	167	47 (41.2%)	59 (41.5%)	61 (55.0%)	0.0397
Never	200	67 (58.8%)	83 (58.5%)	50 (45.0%)	
HER2					
Pos	22	4 (3.4%)	10 (6.9%)	8 (6.8%)	0.2576
Neg	357	114 (96.6%)	134 (93.1%)	109 (93.2%)	
EBV					
Pos	11	0	0	11 (11.7%)	<0.0001
Neg	278	94 (100%)	101 (100%)	83 (88.3%)	
MSI					
MSI	12	0	3 (3.3%)	9 (10.2%)	0.0013
MSS	251	83 (100%)	89 (96.7%)	79 (89.8%)	
TMB					
TMB‐High	33	7 (8.4%)	9 (9.8%)	17 (19.3%)	0.0304
TMB‐Low	230	76 (91.6%)	83 (90.2%)	71 (80.7%)	

The median age of the study cohort was 58 years (range, 25–86 years), and 249 patients (62%) were men. Among 367 patients assessed for smoking history, 167 (46%) were current or ex‐smokers. Higher PD‐L1 expression (CPS ≥5) was significantly enriched in male patients (*p* = 0.0110) and current or ex‐smokers (*p* = 0.0397). Interestingly, tumors located in the stomach fundus and cardia tended to have higher PD‐L1 expression (Table S2), and none of them were HER2‐positive (Table S3). There were no associations between CPS and age (*p* = 0.1333) or HER2 status (*p* = 0.2576), while EBV positivity (*p* < 0.0001), MSI (*p* = 0.0013), and high TMB (*p* = 0.0304) were significantly associated with increased PD‐L1 expression. There was no difference in survival between patients grouped according to CPS, as shown in Figure S1.

### 3.2 Molecular characteristics of PD‐L1 (+) GC according to NGS

Results from the panel sequencing of 523 cancer‐related genes were available for 241 patients. In all, the most frequently mutated gene was *TP53* (57.3%), and mutations in *FAT1* (26.1%), *ARID1A* (25.3%), *NOTCH3* (24.5%), *HIST1H1C* (24.5%), *LRP1B* (24.1%), *ARID1B* (23.7%), *CREBBP* (22.4%), *BRCA2* (22.0%), *SPTA1* (21.2%), and *MDC1* (20.3%) were frequently detected. The most frequently amplified gene was *ERBB2* (10.4%), followed by *RICTOR* (9.5%), *CCNE1* (9.1%), *MYC* (9.1%), *FGFR2* (5.0%), *FGF7* (4.6%), and *EGFR* (4.1%). Variants with strong (tier I) or potential (tier II) clinical significance were detected for at least one gene in 117 (48.5%) patients, mainly including *ERBB2* (13 with mutations and 25 with amplifications), *KRAS* (18 with mutations and five with amplifications), *PIK3CA* (17 with mutations and five with amplifications), *FGFR2* (one with mutation, 12 with amplifications, and four with fusions), *EGFR* (10 with amplifications), *MET* (six with amplifications and three with fusions), and *BRCA2* (nine with mutations). Tumors with at least one tier I/II variant demonstrated significantly higher PD‐L1 expression than those without any tier I/II variants (Figure S2).

**FIGURE 2:**
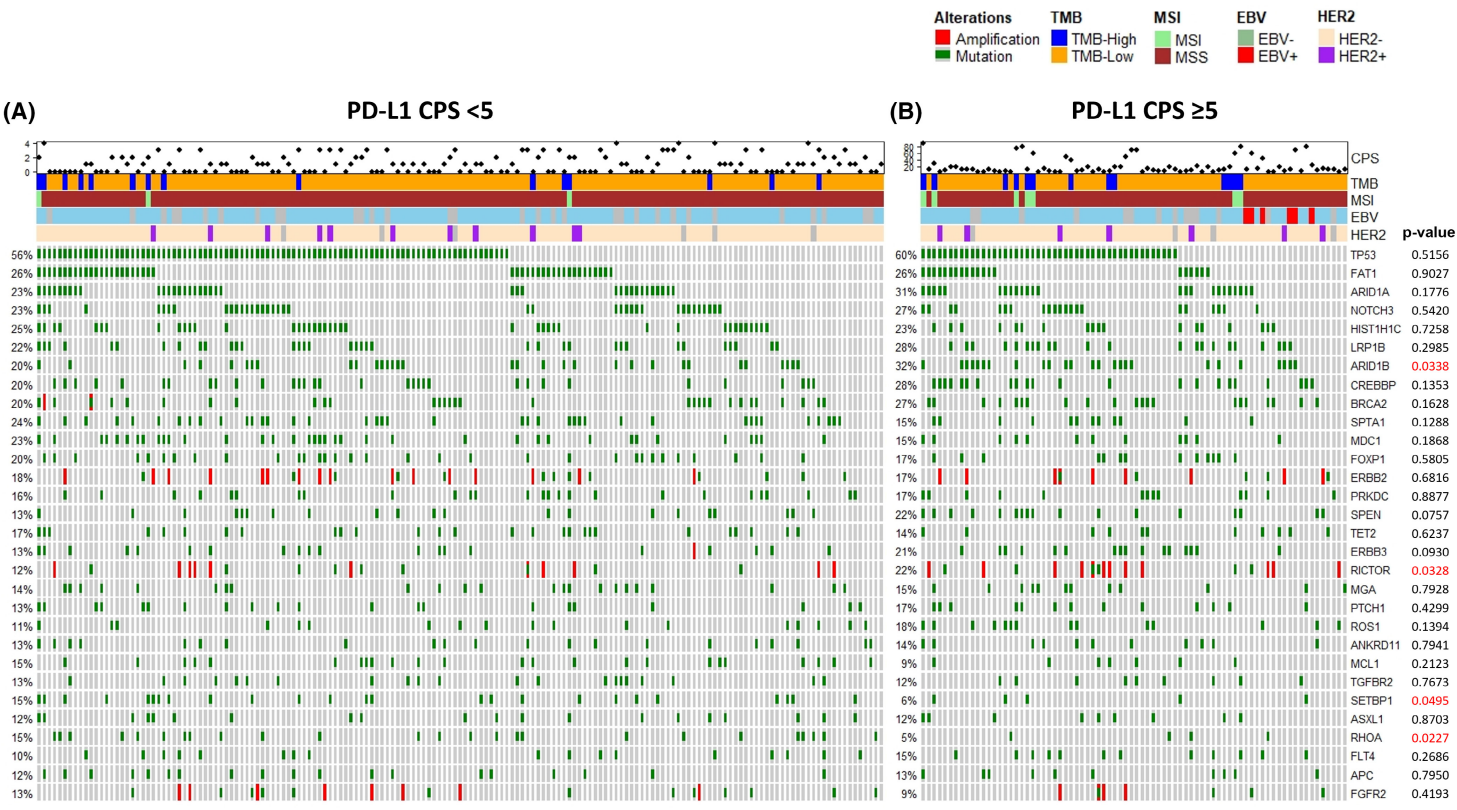
Genomic profiling of (A) PD‐L1 CPS <5 (*n* = 163) and (B) PD‐L1 CPS ≥5 (*n* = 78) GC tumors. CPS (log‐scale), TMB, MSI, EBV, and HER2 statuses are shown at the top. Gene alterations with frequencies and *p*‐value from the chi‐square test between two groups are shown below.

Next, we compared the frequency of genomic alterations between patients with GC with PD‐L1 CPS <5 (*n* = 163) and those with CPS ≥5 (*n* = 78) (Figure 2 and Table S4). In patients with PD‐L1 CPS ≥5 GC, mutations in *ARID1B* (*p* = 0.0338), *BARD1* (*p* = 0.0419), *PIK3CA* (*p* = 0.0067), *SLIT2* (*p* = 0.0402), *CARD11* (*p* = 0.0239), *HSP90AA1* (*p* = 0.0346), *TET1* (*p* = 0.0013), *GLI1* (*p* = 0.0092), *DICER1* (*p* = 0.0233), *PPM1D* (*p* = 0.0233), *ETV6* (*p* = 0.0377), *GEN1* (*p* = 0.0377), *NF2* (*p* = 0.0377), and *HGF* (*p* = 0.0330) were significantly more frequent in comparison with patients with low PD‐L1 GC. In the PD‐L1 low group (CPS <5), mutations in *SETBP1* (*p* = 0.0495), *RHOA* (*p* = 0.0227), and *TCF3* (*p* = 0.0489) were significantly enriched. Notably, amplifications in *RICTOR* (*p* = 0.0328), *MYC* (*p* = 0.0049), *CDK6* (*p* = 0.0377), and *MET* (*p* = 0.0145) were significantly more frequent in the PD‐L1 high (CPS ≥5) GC patient cohort.

**FIGURE 3:**
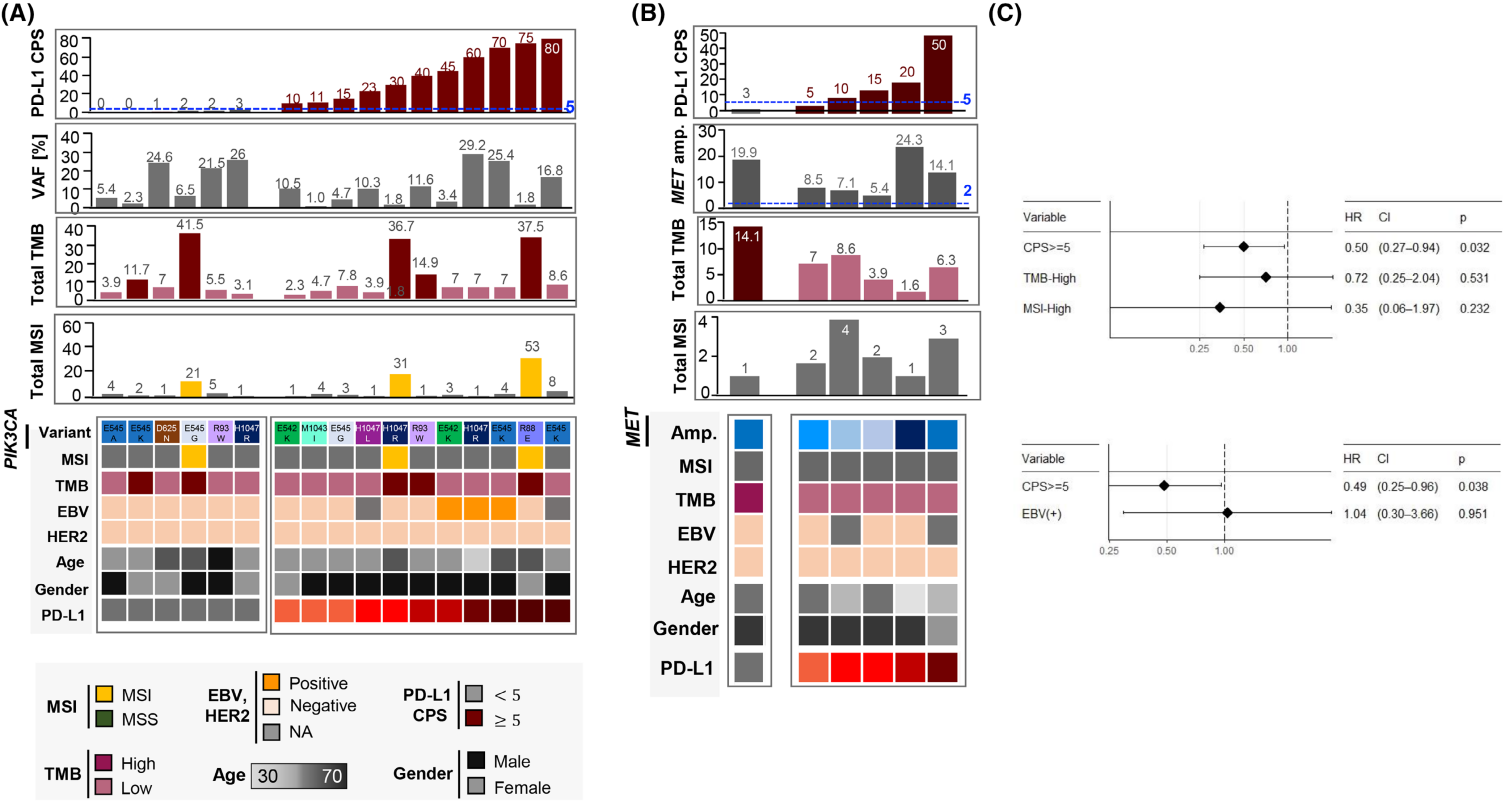
Genomic features of patients with GC with (A) *PIK3CA* mutation or (B) *MET* amplification. (C) Multivariable models for PFS (*n* = 62 for TMB and MSI; *n* = 61 for EBV). HR, hazard ratio; CI, confidence interval with 95% confidence.

Moreover, we investigated the molecular characteristics of GC tumors with *PIK3CA* mutations or *MET* amplifications, given that the treatment arms assigned by these biomarkers demonstrated the highest response rates in the VIKTORY trial.^10^ Both *PIK3CA* mutation and *MET* amplification were significantly enriched in patients with CPS ≥5 as described above. Among 17 patients with *PIK3CA* mutation, 15 (88%), 11 (65%), and eight (47%) patients had PD‐L1 CPS ≥1, ≥5, and ≥ 20, respectively (Figure 3A). Among six patients with *MET* amplification, six (100%), five (83%), and two (33%) patients had PD‐L1 CPS ≥1, ≥5, and ≥ 20, respectively (Figure 3B). Additionally, two patients with *PIK3CA* mutations had CPS <5 but had TMB‐high tumors, and one patient with *MET* amplification had CPS <5 but also had TMB‐high tumors. Taken together, patients selected based on *PIK3CA* mutation and *MET* amplification are promising candidates for ICI therapy.

### 3.3 Impact of PD‐L1 positivity on response to ICI

We identified 108 patients who received ICI therapy, of whom 89 were evaluated for ICI response. For a sample size of 89, the expected effect size for a 2 × 2 chi‐squared test was 0.297, which approximates the conventional medium effect size of 0.3, at a statistical power of 0.8, and significance level of 0.05. The ORR for ICI treatment increased with CPS as follows: 19% for CPS <1, 30% for 1 ≤ CPS <5, and 49% for CPS ≥5 (Table S5). CPS ≥5 was significantly associated with response to ICI (*p* = 0.0285), compared to an ORR of 26% in patients with CPS <5 (Table 2). ORR was higher in EBV‐positive (50%, *n* = 6) and MSI (57%, *n* = 7) patients. Notably, high TMB was significantly associated with response to ICI (*p* = 0.0157), with an ORR of 73% for patients with high TMB (*n* = 11). However, in multivariate analyses, only CPS retained statistically significant (*p* = 0.032 and 0.038) associations with PFS in ICI‐treated patients, whereas that of TMB, MSI, and EBV positivity with CPS were not significant (Figure 3C). In terms of OS, none of the PD‐L1 CPS, TMB, MSI, or EBV positivity values showed a statistically significant association in the multivariable analyses (Figure S3). Among the 399 patients, 387 patients received palliative 1 L chemotherapy, and 353 patients were evaluated for the best treatment response. ORR for 1 L chemotherapy was 34.6% for CPS <1, 53.2% for 1 ≤ CPS <5, and 46.7% for CPS ≥5, which were not positively correlated (Table S5). In addition, patients with CPS ≥5 showed trends of favorable OS after 1 L and 2 L cytotoxic chemotherapy, but this trend was not observed for PFS (Figure S4), indicating the impact of subsequent ICI therapy on OS. Taken together, these results demonstrate that PD‐L1 expression is significantly associated with the response to ICIs but not with cytotoxic chemotherapy.

**TABLE 2:** Associations between molecular features and response to ICI therapy.

Feature	Sample size, *n*	CR/PR, *n* (%)	SD/PD, *n* (%)	*p* Value (χ2 test)	*p* Value (Fisher exact test)
All	89	31 (34.8%)	58 (65.2%)		
PD‐L1					
CPS ≥5	35	17 (48.6%)	18 (51.4%)	0.0285	0.0403
CPS <5	54	14 (25.9%)	40 (74.1%)		
EBV					
Pos	6	3 (50%)	3 (50%)	0.5128	0.6637
Neg	55	20 (36.4%)	35 (63.6%)		
MSI					
MSI	7	4 (57.1%)	3 (42.9%)	0.3354	0.4252
MSS	55	21 (38.2%)	34 (61.8%)		
TMB					
TMB‐High	11	8 (72.7%)	3 (27.3%)	0.0157	0.0211
TMB‐Low	51	17 (33.3%)	34 (66.7%)		

**FIGURE 4:**
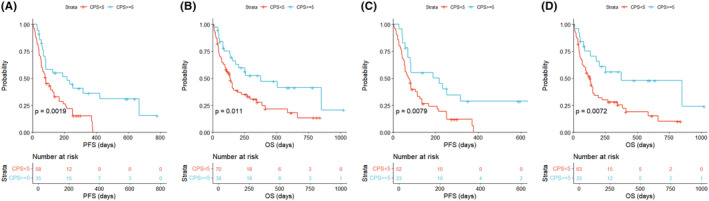
Survival according to PD‐L1 score to anti‐PD1 therapy (A‐B) in all patients treated with ICIs and (C‐D) after excluding EBV‐positive, MSI, or TMB‐high patients. *p*‐value was obtained from the log‐rank test.

When patients were stratified by CPS ≥5 versus CPS <5, there were significant differences in both PFS (*p* = 0.0019) and OS (*p* = 0.011) for overall patients (Figure 4A,B). Survival outcomes were significantly associated with CPS, even when EBV‐positive, MSI, or high TMB patients were excluded (*p* = 0.0079 for PFS and *p* = 0.0072 for OS; Figure 4C,D). In addition, we analyzed previously reported data from a phase 2 clinical trial of pembrolizumab monotherapy in patients with metastatic GC.^7^ For patients confirmed to be EBV‐negative with MSS (*n* = 39), the DCR was 39% for CPS <1, 71% for 1 ≤ CPS <5, and 100% for CPS ≥5, which was significant for its association with CPS (*p* = 0.0271; Table S6).

Lastly, we investigated the association between genomic alterations and response to ICIs (Table S7). The NGS results were reported for 60 patients who were evaluated for ICI treatment responses. Mutations in *TET2* (*p* = 0.0088), *IRS2* (*p* = 0.0173), *DOT1L* (*p* = 0.0259), *PTPRT* (*p* = 0.0259), and *LRP1B* (*p* = 0.0484) were significantly associated with a high ORR (62.5%–100%), while mutation in *MDC1* (*p* = 0.0455) was significantly associated with a low ORR (22.2%). When these gene mutations, together with PD‐L1 CPS, EBV, MSI, and TMB, were subjected to stepwise feature selection for the logistic regression model predicting the ICI response, the following variables were selected: PD‐L1 CPS and mutations in *PTPRT*, *TET2*, *DOT1L*, and *MDC1*. The AUC value of this model was 0.851, and there were significant survival differences (*p* = 0.03 for PFS, *p* = 0.046 for OS) according to the predicted response probability (Figure S5).

## 4 DISCUSSION

In this study, we investigated the clinical significance of PD‐L1 expression and its association with other molecular characteristics in 399 patients with metastatic GC who were consecutively treated at a single center. The percentage of PD‐L1 CPS ≥5 in real‐world data was 30%, which correlated with previous results.^19^, ^20^, ^21^ In addition, using the PD‐L1 assay, we identified that 28 patients had a known etiology for “inflamed” tumors with features such as EBV positivity, MSI, or high TMB. At least 48 patients were confirmed to not have EBV positivity, MSI, or high TMB but still had PD‐L1 CPS ≥5. Given the higher proportion (41%) of PD‐L1 CPS ≥5 in tumors in the upper region of the stomach and that none of them were HER2‐positive, ICIs could specifically benefit these patients. PD‐L1 expression was not associated with OS, which agrees with previous results from late‐stage patients in the Asian Cancer Research Group cohort^22^ and another cohort of Korean and US patients.^23^ PD‐L1 expression was not associated with response to cytotoxic chemotherapy, but was associated with response to ICIs. Favorable responders to ICIs were enriched in patients with CPS ≥5 (49%), EBV (50%), MSI (57%), and TMB‐high (73%), which is in line with the results from a clinical trial of pembrolizumab monotherapy.^7^ Importantly, when patients with high EBV, MSI, or TMB were excluded, CPS was significantly associated with PFS and OS. Taken together, although there are correlations between the PD‐L1 CPS and EBV/MSI/TMB, a comprehensive assessment of these biomarkers may be required to maximize the benefits of ICI treatment.

In this study, we identified several gene alterations that were significantly associated with PD‐L1 expression, including clinically actionable targets, such as *PIK3CA* mutation and *MET* amplification. Recent studies on GC have demonstrated the benefit of combining ICIs with other targeted agents, such as trastuzumab (HER2 inhibitor) and ramucirumab (VEGFR2 inhibitor)^4^, ^5^, ^24^; combining ICIs with other targeted agents might also be promising, especially if there is a correlation between CPS and the target biomarker. Particularly, the VIKTORY umbrella trial demonstrated that drugs targeting *PIK3CA* mutations and *MET* amplification showed higher response rates than other targeted agents.^10^ In addition, considering the unfavorable prognosis of GC with *MET* amplification,^25^, ^26^ its therapeutic targeting is an unmet clinical need. The association between *PIK3CA* mutations and PD‐L1 expression is consistent with the enrichment of *PIK3CA* mutations in EBV‐associated GCs,^8^ which have demonstrated favorable responses to ICIs.^7^, ^27^ It is also notable that all patients with either *PIK3CA* mutation or *MET* amplification were negative for HER2 overexpression in our cohort, and therefore required therapeutic options other than HER2 blockade. Furthermore, therapeutic targeting of *RICTOR* and *MYC* amplifications, which were significantly enriched in tumors with PD‐L1 CPS ≥5, previously demonstrated synergistic effects in several cancer types other than GC.^28^, ^29^, ^30^, ^31^ By contrast, tumors with a PD‐L1 CPS <5 were significantly enriched in RHOA mutations, which were previously associated with genomically stable and diffuse subtypes,^8^, ^32^ requiring further development of treatment strategies for these patients. Collectively, we believe that combining ICIs with other targeted agents would be particularly beneficial for select patients.

We identified six gene alterations that were significantly associated with the response to ICI; five of these were associated with a favorable response, and the sixth was associated with an unfavorable response. The ORR was 62.5%–100% for patients with mutations in *TET2*, *IRS2*, *DOT1L*, *PTPRT*, or *LRP1B*, whereas the ORR was 22.2% for patients with an *MDC1* mutation. Mutations in *TET2*, *PTPRT*, and *LRP1B* are associated with favorable responses to ICIs in other cancer types.^33^, ^34^, ^35^ In GC, *LRP1B* mutation has been shown to be positively correlated with TMB.^36^ These studies support our findings, and further investigations are warranted to determine the clinical significance and underlying biology of these genes in cancer immunity. The addition of these gene mutations to the PD‐L1 CPS resulted in a more accurate prediction model for ICI response in our cohort, which needs to be validated. The reason for one (*PIK3CA* mutation) or no genetic alterations associated with ICI response in previous studies on GC may be due to relatively low ORR (<20%) and small size of the gene panel (<500).^15^, ^16^

This study has some limitations. First, this was a retrospective single‐center study, which requires further validation in prospective multicenter studies. Secondly, the limited sample size may have affected our ability to appropriately test the significance of some molecular features. Although MSI was not significantly associated with response to ICI in our cohort, it is a well‐recognized biomarker of ICI therapy. Moreover, its AUC value was similar to that of TMB (Figure S6), suggesting that the choice of the MSI threshold could increase its predictive accuracy for ICI responses. Although EBV also failed to achieve significance, it is regarded as a promising biomarker for ICI treatment, considering its association with PD‐L1 overexpression and immune cell signaling.^8^ In addition, the prevalence of *PIK3CA* mutations in EBV‐positive GCs may support dual targeting of *PIK3CA* mutations and PD‐1 inhibition. Finally, the intrapatient heterogeneity of molecular characteristics was not considered in our study. It was reported that the concordance of PD‐L1 expression and TMB is approximately 60%–70% between primary tumors versus metastases or before versus after chemotherapy.^37^ Notably, PD‐L1 positivity was 4% and 52% in the paired baseline and post‐treatment samples, respectively, in the VIKTORY trial.^10^ Similarly, 25% of patients treated with targeted therapies experienced PD‐L1 upregulation in the PANGEA trial.^38^ This is in accordance with the notion that targeted agents enhance the immune response to tumor antigens^5^ and their combination with ICI may demonstrate a synergistic effect.

In conclusion, the selection of patients who are expected to benefit from ICI treatment requires comprehensive testing of PD‐L1, EBV, MSI, and TMB. Assessment of a single biomarker is insufficient to guide ICI therapy. The application of NGS not only provides a rationale for the use of targeted agents but also enables the identification of more candidates for ICI treatment. We believe that the combination of ICIs and targeted agents with chemotherapy may further improve the outcomes in patients with GC. Although many recent clinical trials of targeted agents have demonstrated negative results in GC,^39^ further investigations into the combination of these drugs with ICIs are warranted. Indeed, the combination of ICI with MET inhibitors, mTOR inhibitors, or BCL2 inhibitors has demonstrated efficacy in several cancer types other than GC.^28^, ^29^, ^31^, ^40^, ^41^, ^42^, ^43^ In addition, there has been a case report of a CR to dual anti‐EGFR and anti‐PD‐1 therapies in a patient with chemorefractory GC with EGFR amplification.^44^ The integration of these biomarkers is essential for future trials involving individualized treatments.

## AUTHOR CONTRIBUTIONS

**Minkyue Shin:** Data curation (equal); formal analysis (equal); investigation (equal); methodology (equal); visualization (equal); writing – original draft (equal); writing – review and editing (equal). **Soomin Ahn:** Resources (equal); visualization (equal). **Jaeyun Jung:** Visualization (equal). **Sujin Hyung:** Visualization (equal). **Kyoung Mee Kim:** Resources (equal). **Seung Tae Kim:** Resources (equal). **Won Ki Kang:** Resources (equal). **Jeeyun Lee:** Conceptualization (equal); data curation (equal); funding acquisition (equal); resources (equal); supervision (equal); writing – review and editing (equal).

## FUNDING INFORMATION

This research was supported by a grant from the Korea Health Technology R&D Project through the Korea Health Industry Development Institute (KHIDI), funded by the Ministry of Health and Welfare, Republic of Korea (grant number: HR20C0025) and SKKU Excellence in Research Award Research Fund of Sungkyunkwan University (2022).

## CONFLICT OF INTEREST STATEMENT

The authors declare that this study was conducted in the absence of any commercial or financial relationships that could be construed as potential conflicts of interest.

## Supporting information


Appendix S1

## Data Availability

The datasets generated for this study are available on request to the corresponding author.
