# Supplementary material for: Impact of programmed death‐ligand 1 (PD‐L1) positivity on clinical and molecular features of patients with metastatic gastric cancer
Source: Cancer Med. 2023 Sep 1;12(18):18633–42. doi: 10.1002/cam4.6472 (PMC10557860; doi:10.1002/cam4.6472)
Supplement: Supplementary file 1 — Appendix S1 [file CAM4-12-18633-s001.docx]

***Supplementary Material***

**Impact of programmed death-ligand 1 (PD-L1) positivity on clinical and molecular features of patients with metastatic gastric cancer**

Minkyue Shin, MD, Soomin Ahn, MD, Jaeyun Jung, Ph.D, Sujin Hyung, Ph.D, Kyoung-Mee Kim, MD, Seung Tae Kim, MD, Won Ki Kang, MD, Jeeyun Lee, MD*

*** Correspondence: Jeeyun Lee, M.D.** [jyunlee@skku.edu](mailto:jyunlee@skku.edu)

**Supplementary Figures and Tables**

**
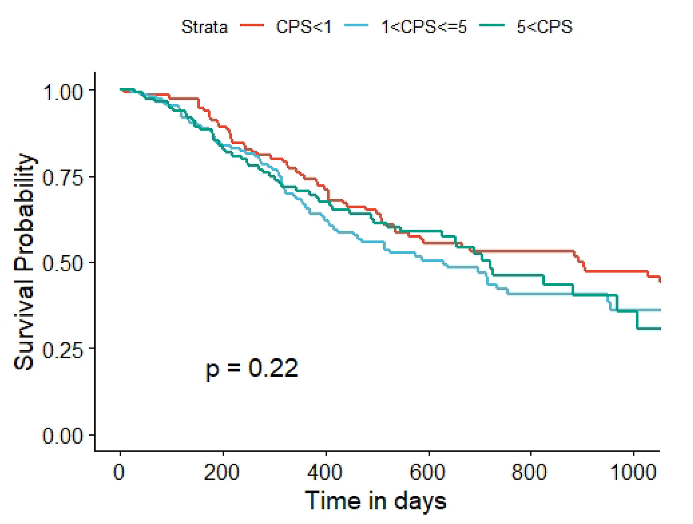
**

**Figure S1.** Overall survival according to PD-L1 score (n = 399). P-value was obtained from the log-rank test.


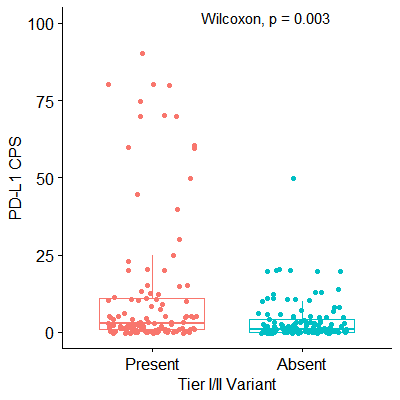


**Figure S2.** PD-L1 CPS according to presence of at least one variant with tier I/II clinical significance (n = 241).


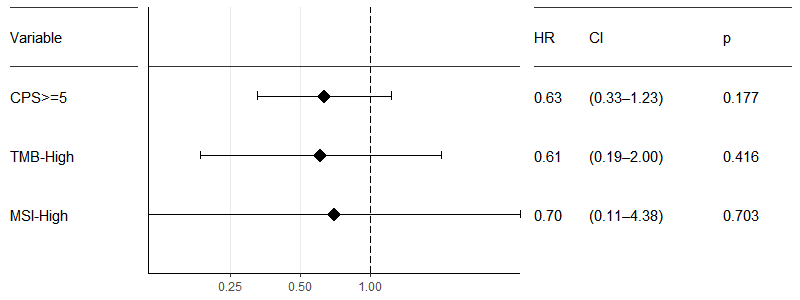


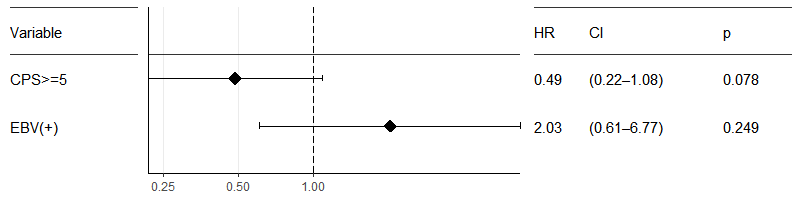


**Figure S3.** Multivariable models for OS (n = 73 for TMB and MSI; n = 71 for EBV). HR, hazard ratio; CI, confidence interval with 95% confidence.


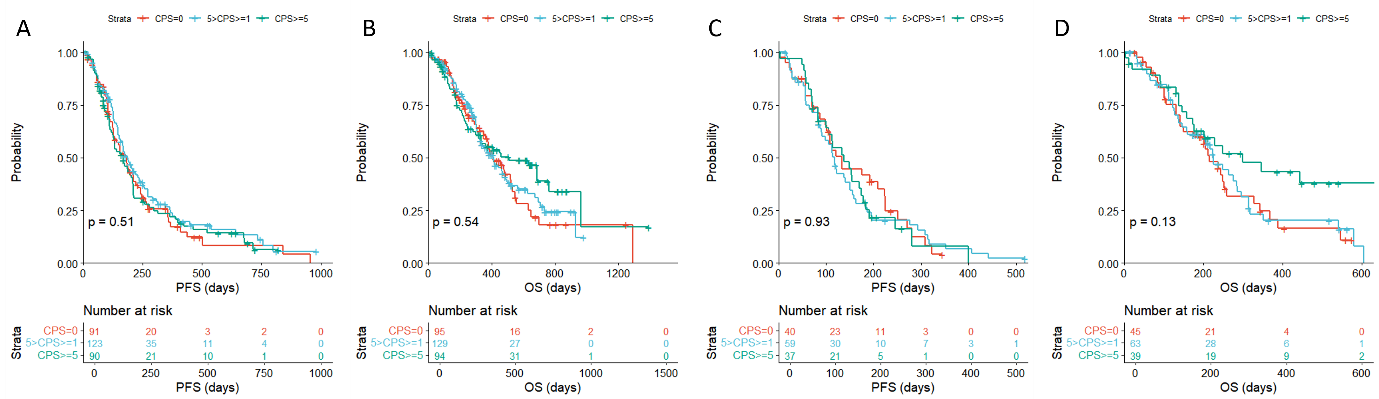


**Figure S4.** Survival according to PD-L1 score after (A-B) the first-line cytotoxic chemotherapy and (C-D) the second-line cytotoxic chemotherapy. P-value was obtained from the log-rank test.


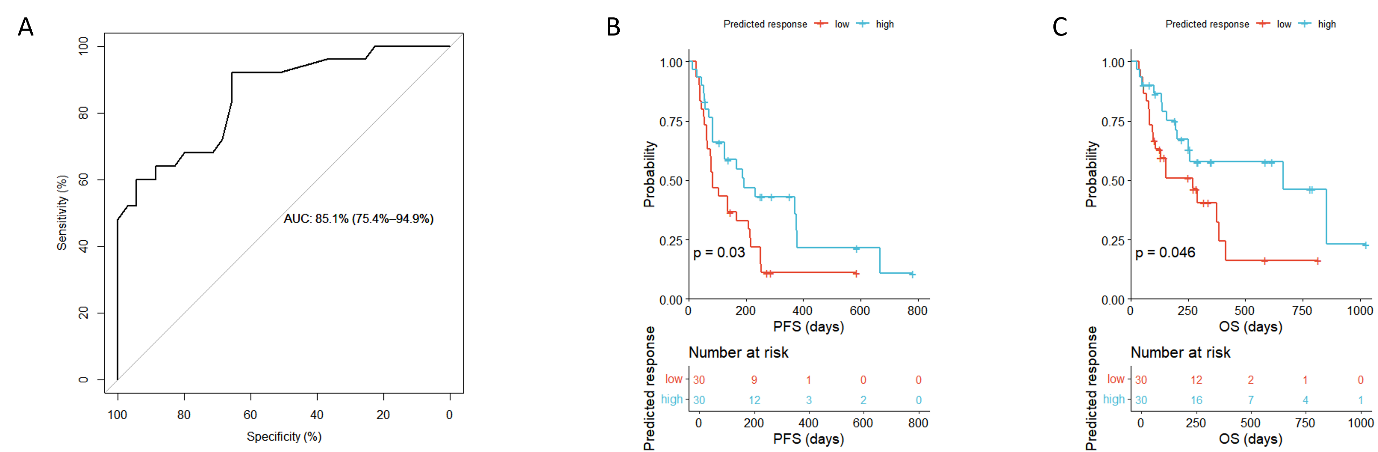


**Figure S5.** (A) Receiver operating characteristic (ROC) curve for the prediction of ICI response from a logistic regression model combining PD-L1 CPS, mutations of *PTPRT, TET2, DOT1L*, and *MDC1*. AUC, Area under the ROC curve. (B-C) Survival according to response probability from the prediction model.


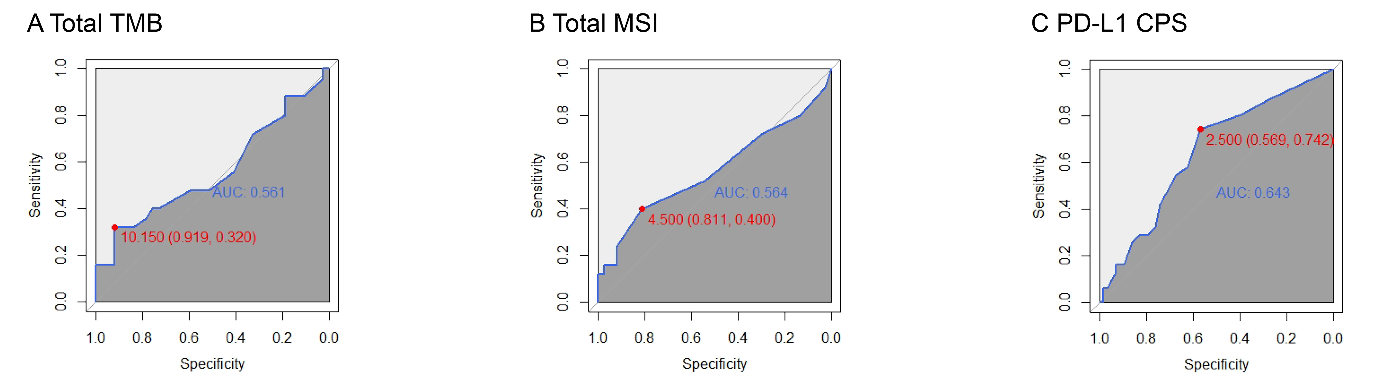


**Figure S6.** Receiver operating characteristic (ROC) curves for the prediction of ICI response from (A) total TMB, (B) total MSI, or (C) PD-L1 expression. Red point refers to the threshold when the sum of sensitivity and specificity is maximum. AUC, Area under the ROC curve.

**Table S1.** First line chemotherapy regimens.

| **Regimen** | **Number of patients** |
| --- | --- |
| Oxaliplatin + Capecitabine | 277 |
| Ramucirumab + Paclitaxel | 22 |
| Cisplatin + S-1/Capecitabine | 20 |
| Pembrolizumab + Oxaliplatin + Capecitabine | 20 |
| Trastuzumab + Capecitabine + Cisplatin/Oxaliplatin | 14 |
| Leucovorin + Fluorouracil + Oxaliplatin | 13 |
| Nivolumab + Oxaliplatin + Capecitabine | 9 |
| Paclitaxel | 8 |
| Pembrolizumab + Trastuzumab + Oxaliplatin + Capecitabine | 2 |
| ZW25 + Tislelizumab + Oxaliplatin + Capecitabine | 1 |
| Nivolumab | 1 |

**Table S2. Association of tumor biopsy sites with PD-L1 expression**

| **Anatomic region** | **CPS <1** | **1≤ CPS <5** | **5≤ CPS** |
| --- | --- | --- | --- |
| Stomach, fundus or cardia | 9 | 14 | 16 |
| Stomach, body | 55 | 75 | 55 |
| Stomach, antrum or pylorus | 37 | 46 | 38 |
| Stomach, involving more than two of above | 10 | 13 | 8 |
| Stomach, anastomosis or unspecified site | 6 | 3 | 1 |
| Distant metastatic site | 5 | 4 | 4 |

**Table S3. Association of tumor biopsy sites with HER2 expression**

| **Anatomic region** | **HER2 negative** | **HER2 positive** |
| --- | --- | --- |
| Stomach, fundus or cardia | 35 | 0 |
| Stomach, body | 165 | 11 |
| Stomach, antrum or pylorus | 111 | 5 |
| Stomach, involving more than two of above | 28 | 3 |
| Stomach, anastomosis or unspecified site | 8 | 1 |
| Distant metastatic site | 10 | 2 |

**Table S4.** Gene alterations significantly (P < 0.05) associated with PD-L1 expression.

|  | CPS <5, n (%) | 5≤ CPS, n (%) | P value  (χ2 test) | P value  (Fisher exact test) |
| --- | --- | --- | --- | --- |
| **Mutation** |  |  |  |  |
| ARID1B | 32 (19.6%) | 25 (32.1%) | 0.0338 | 0.0368 |
| SETBP1 | 25 (15.3%) | 5 (6.4%) | 0.0495 | 0.0602 |
| RHOA | 25 (15.3%) | 4 (5.1%) | 0.0227 | 0.0325 |
| BARD1 | 13 (8.0%) | 13 (16.7%) | 0.0419 | 0.0481 |
| PIK3CA | 7 (4.3%) | 11 (14.1%) | 0.0067 | 0.0156 |
| TCF3 | 19 (11.7%) | 3 (3.8%) | 0.0489 | 0.0565 |
| SLIT2 | 10 (6.1%) | 11 (14.1%) | 0.0402 | 0.0510 |
| CARD11 | 9 (5.5%) | 11 (14.1%) | 0.0239 | 0.0427 |
| HSP90AA1 | 7 (4.3%) | 9 (11.5%) | 0.0346 | 0.0504 |
| TET1 | 4 (2.5%) | 10 (12.8%) | 0.0013 | 0.0024 |
| GLI1 | 4 (2.5%) | 8 (10.3%) | 0.0092 | 0.0214 |
| DICER1 | 4 (2.5%) | 7 (9.0%) | 0.0233 | 0.0418 |
| PPM1D | 4 (2.5%) | 7 (9.0%) | 0.0233 | 0.0418 |
| ETV6 | 2 (1.2%) | 5 (6.4%) | 0.0250 | 0.0377 |
| GEN1 | 2 (1.2%) | 5 (6.4%) | 0.0250 | 0.0377 |
| NF2 | 2 (1.2%) | 5 (6.4%) | 0.0250 | 0.0377 |
| HGF | 0 | 3 (3.8%) | 0.0117 | 0.0330 |
| **Amplification** |  |  |  |  |
| RICTOR | 11 (6.7%) | 12 (15.4%) | 0.0328 | 0.0581 |
| MYC | 9 (5.5%) | 13 (16.7%) | 0.0049 | 0.0078 |
| CDK6 | 2 (1.2%) | 5 (6.4%) | 0.0250 | 0.0377 |
| MET | 1 (0.6%) | 5 (6.4%) | 0.0069 | 0.0145 |

**Table S5.** Association of PD-L1 expression with treatment responses. P-value was obtained from the Cochran-Armitage trend test.

| Best response to | Sample size, n | CPS <1, n (%) | 1≤ CPS <5, n (%) | 5≤ CPS, n (%) | p-value |
| --- | --- | --- | --- | --- | --- |
| **1st Chemotherapy** |  |  |  |  |  |
| CR/PR | 161 | 37 (34.3%) | 74 (53.2%) | 50 (47.2%) | 0.0562 |
| SD/PD | 192 | 71 (65.7%) | 65 (46.8%) | 56 (52.8%) |  |
| **Immune checkpoint inhibitor** |  |  |  |  |  |
| CR/PR | 31 | 4 (19.0%) | 10 (30.3%) | 17 (48.6%) | 0.0201 |
| SD/PD | 58 | 17 (81.0%) | 23 (69.7%) | 18 (51.4%) |  |

**Table S6.** Association of best response to Pembrolizumab with PD-L1 expression in MSS, EBV(-) patients (n=39). P-value was obtained from the Cochran-Armitage trend test.

| Best response | | Sample size, n | | CPS <1, n (%) | 1≤ CPS <5, n (%) | 5≤ CPS, n (%) | p-value |
| --- | --- | --- | --- | --- | --- | --- | --- |
| CR/PR/SD | 21 | | 9 (39.1%) | | 10 (71.4%) | 2 (100%) | 0.0271 |
| PD | 18 | | 14 (60.9%) | | 4 (28.6%) | 0 |  |

**Table S7.** Gene alterations significantly (P < 0.05) associated with best response to ICI.

| ICI treated patients | Sample size, n | CR/PR, n (%) | SD/PD, n (%) | P value (χ2 test) | P value  (Fisher exact test) |
| --- | --- | --- | --- | --- | --- |
| **TET2** |  |  |  |  |  |
| Mutation | 12 | 9 (75%) | 3 (25%) | 0.0088 | 0.0191 |
| Wild-type | 48 | 16 (33.3%) | 32 (66.7%) |  |  |
| **IRS2** |  |  |  |  |  |
| Mutation | 7 | 6 (85.7%) | 1 (14.3%) | 0.0119 | 0.0173 |
| Wild-type | 53 | 19 (35.8%) | 34 (64.2%) |  |  |
| **DOT1L** |  |  |  |  |  |
| Mutation | 4 | 4 (100%) | 0 (0%) | 0.0143 | 0.0259 |
| Wild-type | 56 | 21 (37.5%) | 35 (62.5%) |  |  |
| **PTPRT** |  |  |  |  |  |
| Mutation | 4 | 4 (100%) | 0 (0%) | 0.0143 | 0.0259 |
| Wild-type | 56 | 21 (37.5%) | 35 (62.5%) |  |  |
| **MDC1** |  |  |  |  |  |
| Mutation | 18 | 4 (22.2%) | 14 (77.8%) | 0.0455 | 0.0525 |
| Wild-type | 42 | 21 (50%) | 21 (50%) |  |  |
| **LRP1B** |  |  |  |  |  |
| Mutation | 16 | 10 (62.5%) | 6 (37.5%) | 0.0484 | 0.0751 |
| Wild-type | 44 | 15 (34.1%) | 29 (65.9%) |  |  |
